# Concerns regarding Covid-19 vaccine certificates

**DOI:** 10.1017/pls.2020.29

**Published:** 2021-01-11

**Authors:** Brian R. Spisak, Eric J. McNulty

**Affiliations:** 1 Harvard University; University of Otago; 2 Harvard University

**Keywords:** Covid-19 Vaccine Certificates, Intrinsic Motivation, Prosocial Behavior, Leadership

## Abstract

Besides vaccine certificates, research suggests leaders also need to trigger society’s intrinsic motivation to help in order to achieve lasting and equitable solutions.

There are increasing calls for issuing COVID-19 testing and vaccination status certificates as a way out of broad stay-at-home orders. They are meant to reopen schools, kick-start international travel, and reinvigorate the economy by certifying “safe” people and rapidly isolating those who are infected. The World Economic Forum, for instance, is supporting “CommonPass,” which it describes on its website as a way “to enable people to securely document and present their COVID-19 status (either as test results or an eventual vaccination status) to facilitate international travel and border crossings while keeping health information private” (Whiting & Szabo, 2020).

Done well, such an approach could confer significant benefits, including a rapid transition to the “next normal,” with many more individuals able to socialize and work. Testing and vaccination status certificates are therefore an obvious solution to our troubles and, not surprisingly, advocated in medical journals (Studdert & Hall, 2020) and the popular media (Edlin & Nesbitt, 2020). This is perhaps why large organizations such as the World Economic Forum are actively supporting certification trials (Berger, 2020). These sources claim that certifications are a fair price to pay for our freedoms of movement and commerce.

However, this fair price can come with hidden costs (Phelan, 2020). Done poorly, testing- and vaccination-based regimes could set a precedent for distinct classes of people based on their perceived health risk and access to vaccination. Biases and discrimination against racial minorities, who have been disproportionally affected by COVID-19, could increase (Devakumar et al., 2020). Failure to get a certification could also become a badge of shame (Kim et al., 2011). This could, in turn, trigger attempts to forge certifications or the creation of a subclass of uncertified people employed off the books at lower wages and without benefits.

Such situations worsen inequalities and systemic problems in society. For example, a migrant worker recently infected in South Australia told contact tracers that he had visited a pizza shop briefly to collect a pizza when he was in fact an employee. The lie caused authorities to think the strain was highly contagious, consequently triggering a hard lockdown of 1.7 million residents (Kurmelovs, 2020).

The obvious question is why did this individual lie, and the likely answer is fear. It is known that he had multiple jobs and perhaps was afraid to tell the truth because certain types of visas have employment restrictions. Here one experiences a threat of punishment, thus reducing the intrinsic motivation to help. At scale, this means that millions of people are likely in stressful situations (e.g., fear of job loss, deportation, or social isolation) inhibiting their prosocial tendencies.

The path to avoiding this worst-case scenario and achieving the best case begins with the assumptions underlying the planning process. It is argued that a punishment-centric system, in which failure to achieve certification could come with significant costs, such as job loss, banishment from campus, or deportation, is likely to create inequalities and exacerbate discrimination (Bhandari & Moore, 2020). Unfortunately, rules-based bureaucracies often default to such approaches. The alternative is a prosocial process that emphasizes testing and vaccination while minimizing the tangible and intangible costs of a positive result for COVID-19. Such a strategy would stimulate intrinsic motivations to participate rather than the motivations caused by fear and anger.

Intrinsic motivations are known to have stronger and longer-lasting effects on behavior (Ryan & Deci, 2017), and thus they are more likely to result in the desired restriction-easing outcomes. The key is making people realize that getting tested, taking steps to avoid infection, and eventually getting vaccinated are in everyone’s interest. For example, countering an individual’s motivation to support anti-lockdown protests may require an alternative narrative based on the intrinsic motivation to defend one’s country against COVID-19. New Zealand, for instance, has shown that introducing a drastic and united front, with a narrative based on “a team of five million” and “go hard, go early,” is an effective response for rapidly lifting the restrictions anti-lockdown protestors condemn.

To arrive at this intrinsically motivated outcome, a collaborative, multilevel effort is needed (e.g., McNulty et al., 2018). At the governmental level, effective protocols for individuals testing positive for COVID-19 would supply subsidized quarantine, job security, and other features of a strong social safety net. Such broad and balanced stakeholder orientations are typically associated with intrinsic, prosocial motivations and sustained long-term performance—both socially and economically (Spisak et al., 2015). Governments will also have to cooperate internationally to ensure that vaccinations are not disproportionately stockpiled in wealthier nations (Cuddy, 2020). A globalized world requires globalized support.

As society returns to work, school, and so on, the organizational level will need to create inclusive environments in which vigilant (re)testing and vaccination (not just certifications) are the measurable and formally supported outcomes. The promotion of (re)testing and vaccination in organizations, working in tandem with government-backed safety nets, reinforces norms of reciprocity and trust. For example, an employer’s COVID-19 recovery message might state, “As testing helps keep us all safer, we will guarantee your job and pay should you have to quarantine.” Decades of research demonstrates that groups exhibiting this sort of reciprocity and trust are high performing (Evans & Davis, 2005), whereas unjust punishment based on uncontrollable events—a COVID-19 infection resulting in unpaid quarantine, for example—often leads to an array of organizationally detrimental behaviors (Mooijman & Graham, 2018).

On an interpersonal level, a concern for others is essential for starting and keeping healthy relationships. Compassion and empathy, for instance, are associated with intrinsically motivated helping behavior (Goetz et al., 2010) as well as the reduction of stigma and discrimination (Eisenberg et al., 2010). Interpersonal relationships based on these intrinsic factors will encourage prosocial norms of (re)testing and vaccination, in contrast with what extrinsic certification anxiety offers—such as animosity and disgust (Tybur et al., 2013).

On an intrapersonal level, avoiding maladaptive guilt and shame also requires an emphasis on *supporting* what an individual can actively control, such as (re)testing and vaccination, rather than *punishing* less controllable certification outcomes. Most individuals will eventually break bubbles and risk infection as society reopens. It is therefore essential that a personal sense of power over a controllable outcome—namely, *taking the test and getting vaccinated—*is psychologically internalized as preferable to the fear of punishment. Establishing intrinsic motivation through a sense of personal control can lead to a variety of positive outcomes such as general wellbeing, proactive behavior, better interpersonal relationships, higher levels of organizational satisfaction and performance, as well as a greater sense of civic engagement and responsibility (Caprara & Steca, 2005; Fung, 2015; Judge & Bono, 2001).

These four levels—governmental, organizational, interpersonal, and intrapersonal—are interlocking and require coordinated support from one layer to the next. This sort of systemic approach is essential even if the complexity of the response and the required investment may deter some officials. The desired outcome is far too critical for the injection of simplified certification remedies.

It is also important to note that not every situation will have this full complement of support. Some countries lack social safety nets, some employers do not fully support their employees, and many individuals are socially isolated. These gaps, in short, must be filled. Not doing so will result in stigma, discrimination, counterproductive work environments, and more. Conversely, if gaps are found and filled, then the outcome is a proactive, prosocial, and prepared COVID-19 response.

Responding effectively to COVID-19 is more complex than solving the obvious public health and economic problems with seemingly easy answers. Many interlocking social systems need to synchronize for a viable recovery—from political science and government policy to psychological science and individual behavior. COVID-19 is a multidisciplinary problem requiring a multidisciplinary solution. Business and society must appreciate this complexity to preserve life *and* liberty.
